# Low-Dose Methotrexate Toxicity Presenting With Inguinal Lesions and Pancytopenia: A Case Report

**DOI:** 10.7759/cureus.101001

**Published:** 2026-01-07

**Authors:** Kathleen A Clark, Ashleigh Wilkerson, Mike Zickella, Kaitlyn M Unterman

**Affiliations:** 1 Medical School, Alabama College of Osteopathic Medicine, Dothan, USA; 2 Family Medicine, Mobile Infirmary Medical Center, Mobile, USA

**Keywords:** chronic kidney disease (ckd), inguinal lesion, methotrexate toxicity, oral mucositis, pancytopenia

## Abstract

Methotrexate (MTX) is a folate antagonist widely used as a disease-modifying antirheumatic drug (DMARD). Despite its efficacy and broad therapeutic range, its narrow safety margin and reliance on renal clearance confer risk for severe multisystem toxicity, particularly in patients with renal impairment, comorbidities, or dosing errors.

We describe a 74-year-old male with coronary artery disease, type 2 diabetes, stage 3a chronic kidney disease, obesity, and seronegative rheumatoid arthritis who presented with progressive weakness, painful oral and genital ulcers, and three weeks of watery diarrhea. He was taking MTX 20 mg weekly, divided into morning and evening doses every seven days for three months. On admission, he was hypotensive, pancytopenic, and had acute kidney injury with elevated transaminases. His course was complicated by profuse bleeding from groin ulcers and evidence of systemic mucocutaneous toxicity. Laboratory evaluation confirmed cytopenias, transaminitis, and renal dysfunction, which improved with discontinuation of MTX and supportive therapy. Because life-threatening toxicity from low-dose MTX is rare, this case underscores an uncommon yet important manifestation - early detection of gastrointestinal symptoms, painful cutaneous and inguinal ulcerations, and cytopenias is crucial, even at the smallest doses of antirheumatic agents. Identification is paramount, as progression to late-stage toxicity is often refractory to leucovorin rescue.

## Introduction

Low-dose methotrexate (MTX) is a first-line disease-modifying antirheumatic drug (DMARD) used for conditions such as rheumatoid arthritis, psoriasis, and osteosarcoma. It also has an essential role in cancer chemotherapy and is the treatment of many diverse diseases such as acute lymphocytic leukemia, choriocarcinoma, and non-Hodgkin’s lymphoma [1]. This widely used pharmacological drug is a folate antagonist, impairing DNA, RNA, and protein synthesis. Toxicity occurs when folate depletion or drug accumulation impairs rapidly dividing cells, including bone marrow, gastrointestinal mucosa, and skin, leading to multisystem damage. Life-threatening toxicity from low-dose MTX has a 0.1%-1% occurrence rate per year [2]. Patients of older age, renal impairment, dosing errors, and drug interactions experience a much higher risk of toxicity. Individual differences in drug response can also be attributed to genetic polymorphisms in genes that code for drug-metabolizing enzymes, transporters, and targets.

MTX can be safely administered over a wide dosing range, ranging from 20 mg/m^2^ per week to doses of 33,000 mg/m^2^ [2]. The mechanism of action and metabolism of this drug is critical to understanding the presentation of an elevated MTX concentration that could lead to ineffective rescue by leucovorin. Vomiting and diarrhea shortly after MTX administration have been documented in cases of MTX toxicity, but the majority of patients with renal impairment remain asymptomatic in the early stages and ultimately manifest with nonoliguric renal dysfunction [2]. Without early diagnosis based on urine output, serum creatinine levels, and plasma MTX measurements, patients often present only after several weeks with severe mucositis, profound myelosuppression, and, less frequently, dermatitis [3]. At this symptomatic stage, even high doses of leucovorin rescue are unlikely to effectively reverse MTX toxicity.

## Case presentation

A 74-year-old male with a history of coronary artery disease, type 2 diabetes mellitus, stage 3a chronic kidney disease, gout, intermittent polyarthralgia, melanoma, and morbid obesity (BMI 44 kg/m²) presented to the Emergency Department with complaints of progressive generalized weakness, painful oral and cutaneous lesions, and three weeks of watery diarrhea associated with abdominal pain and decreased appetite. He also reported painful ulcers in the penile and inguinal region.

Seven months prior, the patient developed migratory polyarthritis with swelling of his hands and was diagnosed with seronegative rheumatoid arthritis (negative RF, CCP, and HLA-B27). Initial therapy with a methylprednisolone dose pack provided only partial relief. One month later, persistent symptoms led to the initiation of leflunomide 20 mg daily.

The patient continued to experience unresolved joint swelling and morning stiffness. MTX was prescribed at 15 mg weekly and later increased to 20 mg weekly. However, two months later, he presented to the rheumatology clinic with worsening hand and foot pain, as well as three weeks of watery postprandial diarrhea. At this time, he also noted tender inguinal ulcers. His MTX regimen consisted of MTX sodium 2.5 mg tablets, four tablets with breakfast and four tablets with supper, taken once weekly.

**Figure 1:**
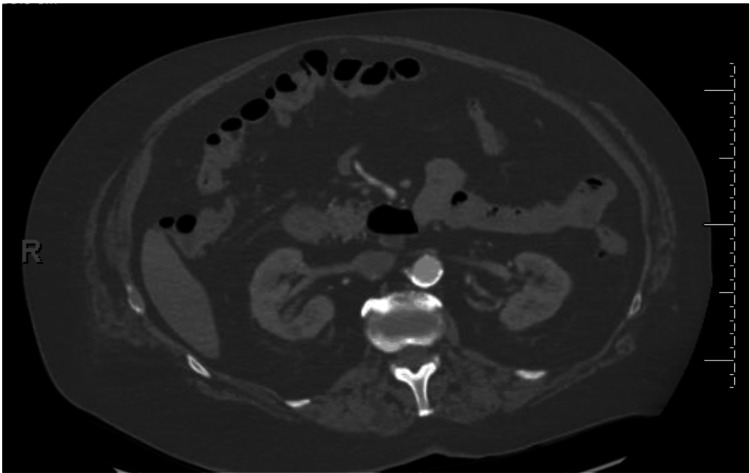
CT abdomen The kidneys and spleen are within normal limits, with no free fluid, free air, or evidence of bowel obstruction.

**Figure 2:**
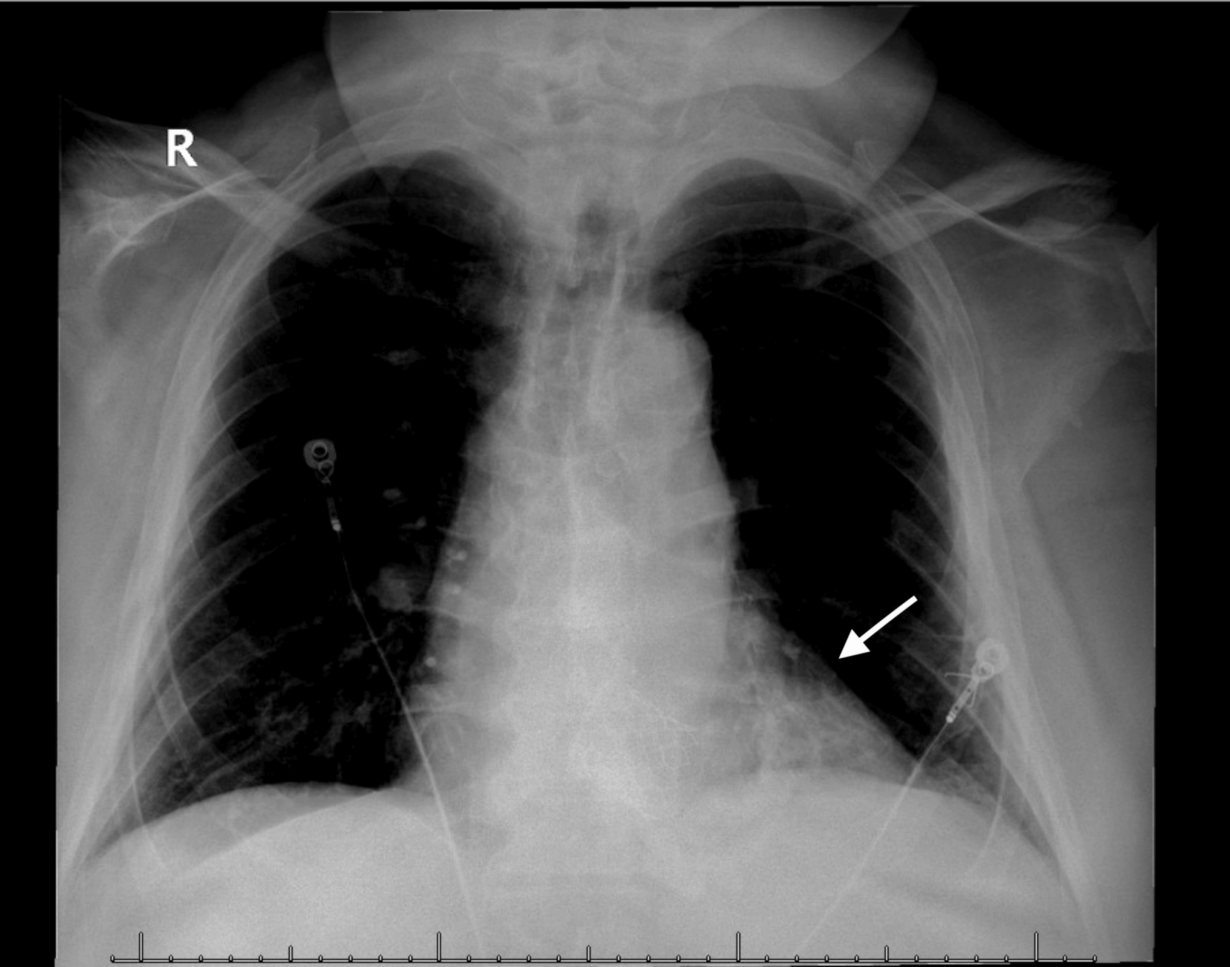
A/P chest X-ray read minimal left basilar atelectasis (arrow)

On arrival at the Emergency Department, he was hypotensive, pancytopenic, and found to have acute kidney injury with elevated transaminases. His groin lesions bled profusely following a procedural incision, requiring local hemostasis with lidocaine and epinephrine. Abdominal imaging demonstrated small bilateral pleural effusions and minimal left basilar atelectasis, while CTA of the abdomen showed no free air or fluid and normal-appearing solid organs (Figures 1-2).

The constellation of mucocutaneous ulcers, diarrhea, cytopenias, renal impairment, and hepatotoxicity raised concern for MTX-induced mucocutaneous and systemic toxicity (Table 1).

**Table 1:** Patient laboratory results AST, Aspartate Aminotransferase; ALT, Alanine Aminotransferase; BUN, Blood Urea Nitrogen; WBC, White Blood Cell; RBC, Red Blood Cell; MCV, Mean Corpuscular Volume; MCH, Mean Corpuscular Hemoglobin

Laboratory Test	Pre-methotrexate	Admission	Discharge	Reference Range
Creatinine (mg/dL)	1.3	1.72	0.96	0.67-1.17
BUN (mg/dL)	51	51	15	5-25
AST (U/L)	32	46	36	0-40
ALT (U/L)	34	52	32	0-40
Glucose (mg/dL)	137	137	131	70-99
WBC (K/uL)	3.3	3.3	8.7	3.5-10.5
RBC (M/uL)	2.50	2.50	2.38	4.32-5.72
Hemoglobin (g/dL)	8.4	8.4	7.9	13.5-17.5
Hematocrit (%)	26.0	26.0	26.4	38.8-50
MCV (fL)	110	104.0	110.9	81.2-95.1
MCH (pg)	33.6	33.6	33.2	26.3-32.9
Platelets (K/uL)	143	143	207	150-450
Absolute Lymphocytes (K/uL)	0.74	0.74	1.80	0.9-2.9

## Discussion

MTX, a folate antagonist and cornerstone DMARD, remains widely prescribed for rheumatoid arthritis, malignancies, and other autoimmune conditions due to its efficacy, affordability, and long-term data [1]. However, its narrow therapeutic index and highly variable pharmacokinetics confer significant risk for systemic toxicity. Toxic effects span multiple organ systems and often present insidiously, requiring a high index of suspicion for timely recognition.

In this case, the patient’s presentation of painful oral and inguinal mucocutaneous ulcers, persistent watery diarrhea, cytopenias, transaminitis, and acute kidney injury is suggestive of MTX-induced multiorgan toxicity. Mucositis is often the earliest manifestation of MTX toxicity, attributable to inhibition of thymidylate and purine synthesis, which impairs DNA replication and disproportionately affects rapidly proliferating epithelial and hematopoietic cells [1]. Gastrointestinal epithelial destruction further explains this patient’s chronic watery diarrhea.

Renal dysfunction played a pivotal role in amplifying toxicity. MTX is predominantly excreted unchanged by glomerular filtration and active tubular secretion. Pre-existing stage 3a chronic kidney disease in this patient would have predisposed to reduced clearance, thereby prolonging systemic exposure [2]. In addition, renal tubular precipitation of MTX and its seven-hydroxy metabolite may exacerbate nephrotoxicity, creating a vicious cycle of impaired clearance and worsening systemic toxicity [4]. The resulting high circulating MTX concentrations further compromise hematopoiesis, hepatic metabolism, and mucosal integrity.

The pancytopenia observed in this patient reflects the well-described myelosuppressive effect of MTX, which results from direct inhibition of DNA synthesis in rapidly dividing bone marrow progenitors. Cytopenias, particularly when accompanied by mucositis, are considered hallmark signs of systemic MTX toxicity [5]. Elevated liver transaminases, although reversible in most cases, point to hepatocellular injury due to intracellular accumulation of MTX polyglutamates within hepatocytes, an effect exacerbated by obesity, diabetes, and pre-existing metabolic stress on the liver.

Notably, the patient’s MTX regimen - eight 2.5 mg tablets split between morning and evening with meals once weekly - raises the possibility of dosing misinterpretation. Accidental daily instead of weekly administration remains one of the most common causes of MTX toxicity, especially in elderly patients with polypharmacy and multiple comorbidities [5]. Even in the absence of confirmed dosing error, complex regimens and lack of patient education are frequent contributors to preventable adverse outcomes.

Taken together, this case emphasizes several well-documented pathways of MTX toxicity-impaired clearance due to renal dysfunction, enhanced tissue accumulation due to obesity and hepatic comorbidity, and possibly pharmacogenetic predisposition. The convergence of these risk factors likely culminated in the patient’s fulminant clinical deterioration.

## Conclusions

MTX toxicity can present rapidly with mucositis, cytopenias, diarrhea, hepatotoxicity, and renal impairment, particularly in patients with underlying kidney disease and multiple comorbidities. Early recognition of oral and cutaneous lesions is critical, as they often lead to systemic involvement. Management begins with immediate drug discontinuation, leucovorin rescue, and aggressive hydration with urine alkalinization. Vigilant monitoring, patient education on weekly dosing, and consideration of pharmacogenetic risk factors remain essential to preventing life-threatening complications.
